# Supplementary material for: Proximal tubule-on-chip as a model for predicting cation transport and drug transporter dynamics
Source: Sci Rep. 2025 Jan 20;15:2580. doi: 10.1038/s41598-025-85653-4 (PMC11747318; doi:10.1038/s41598-025-85653-4)
Supplement: Supplementary file 1 — Supplementary Information. [file 41598_2025_85653_MOESM1_ESM.pdf]

Proximal Tubule-on-Chip as a Model for Predicting Cation Transport and Drug Transporter Dynamics

Isy Petit[1], Quentin Faucher[2], Jean-Sébastien Bernard[1], Perrine Giunchi[1,3,4], Antoine Humeau[1], François-Ludovic Sauvage[1], Pierre Marquet[1,5], Nicolas Védrenne[1,#,*] and Florent Di Meo[1,6,#,*]

[1] P&T, UMR1248, Inserm, Limoges University, Limoges, France.
[2] Division of Pharmacology, Utrecht Institute for Pharmaceutical Sciences, Utrecht University, Utrecht, the Netherlands.
[3] Institut de Recherche en Santé Digestive, Université de Toulouse, INSERM, INRAE, ENVT, Univ Toulouse III, France
[4] Institut de Mécanique des Fluides de Toulouse (IMFT), CNRS & Université de Toulouse, Toulouse, France
[5] Department of Pharmacology, Toxicology and Pharmacovigilance, CHU Limoges, Limoges, France
[6] Université de Limoges, CNRS, Inserm, CHU Limoges, UAR2015, US42, Integrative Biology Health Chemistry and Environment BISCEm, Limoges, France

* These authors contributed equally, # Corresponding authors

Corresponding Authors:

Dr. Florent Di Meo & Dr. Nicolas Védrenne

Inserm U1248 Pharmacology & Transplantation, Univ. Limoges,

2 rue du Prof. Descottes,

87000 F-Limoges, France

florent.di-meo@inserm.fr – nicolas.vedrenne@unilim.fr

Tel: +33(0)5 19 56 42 76

Supplementary Tables

Table S1. List of primers

Gene symbol	References
ABCB1	Hs00184500_m1
ABCC2	Hs00166123_m1
ABCC4	Hs00988717_m1
ABCG2	Hs01053790_m1
SLC22A2	Hs01010726_m1
SLC22A4	Hs00268200_m1
SLC22A5	Hs00929869_m1
SLC22A6	Hs00537914_m1
SLC22A7	Hs00198527_m1
SLC22A8	Hs00188599_m1
SLC47A1	Hs00217320_m1
SLC47A2	Hs00945652_m1
GAPDH	Hs99999905_m1

Table S2. List of antibodies used in the present study.

Target	Reference	Species	Dilution	Fluorescence
**P-glycoprotein**	ab235954	Rabbit polyclonal	1/200.	Na
**Na+/K+ ATPase**	ab210143	Rabbit monoclonal	1/500.	Alexa Fluor® 405
**MATE-1**	ab92295	Goat polyclonal	1/200	Na
**OCT2/SLC22A2**	ab242317	Mouse monoclonal	1/500	Na
**OAT3/SLC22A8**	ab247055	Rabbit polyclonal	1/400	Na
**Secondary antibody**				
**Anti-Rabbit**	A11008	Goat polyclonal	1/1000	Alexa Fluor® 488
**Anti-Goat**	A-11039	Chicken polyclonal	1/1000	Alexa Fluor® 488
**Anti-Mouse**	ab175700	Donkey polyclonal	1/1000	Alexa Fluor® 568

Table S3. Rt-qPCR statistical data

		ABCB1	ABCC2	ABCC4	ABCG2	SLC22A2	SLC22A4	SLC22A5	SLC47A1	SLC47A2
FSS (5 µL/min) compared to rocker-based perfusion condition	Mean	1.81	0.797	0.908	14.461	4.924	0.689	1.179	17.368	16.196
	SD	1.56	1.064	0.936	13.154	6.911	0.397	0.282	16.242	9.844
	SEM	0.637	0.434	0.382	5.37	2.821	0.229	0.163	6.631	5.683
	Adjusted P Value	> 0.99	0.5116	> 0.99	0.0277	> 0.99	0.9457	> 0.99	0.0373	0.1735
	N	6	6	6	6	6	3	3	6	3
FSS (10 µL/min) compared to rocker-based perfusion condition	Mean	2.176	3.761	1.085	5.137	4.074	0.445	1.145	22.744	40.191
	SD	2.527	2.677	1.244	8.232	4.352	0.234	1.083	26.104	7.637
	SEM	0.729	0.892	0.359	2.376	1.451	0.135	0.625	7.536	4.409
	Adjusted P Value	> 0.99	0.5637	> 0.99	> 0.99	0.861	0.102	> 0.99	0.0053	0.0024
	N	12	9	12	12	9	3	3	12	3
FSS (20 µL/min) compared to rocker-based perfusion condition	Mean	3.58	3.318	2.67	26.323	1.907	0.804	1.146	10.785	11.952
	SD	2.255	3.285	1.057	16.964	1.615	0.253	0.814	10.494	5.498
	SEM	1.009	1.469	0.473	7.587	0.724	0.146	0.47	4.693	3.174
	Adjusted P Value	0.0545	> 0.99	0.0706	0.0038	> 0.99	> 0.99	> 0.99	0.0864	0.2226
	N	5	5	5	5	5	3	3	5	3
Metformin [100µM]compared to untreated condition	Mean	3.666	6.299	4.883	1.422	6.423	2.735	2.468	11.412	19.35
	SD	2.29	3.799	1.146	0.696	8.707	3.576	0.443	4.507	7.448
	SEM	1.322	2.193	0.662	0.402	6.157	2.064	0.256	2.602	4.3
	Adjusted P Value	0.1283	0.0073	0.0281	> 0.99	> 0.99	> 0.99	0.0073	0.0117	0.0073
	N	3	3	3	3	3	3	3	3	3
Metformin [100µM] + cimetidine [100µM] compared to untreated condition	Mean	0.649	2.045	1.136	0.381	0.689	0.778	1.149	4.354	2.663
	SD	0.31	0.714	0.252	0.313	0.359	0.54	0.073	3.021	1.29
	SEM	0.179	0.412	0.145	0.181	0.207	0.312	0.042	1.744	0.745
	Adjusted P Value	0.6897	0.2498	> 0.99	0.0911	> 0.99	0.937	0.2498	0.1818	0.2498
	N	3	3	3	3	3	3	3	3	3
Creatinine [10µM] compared to untreated condition	Mean	3.065	3.735	5.652	0.402	3.528	1.401	2.538	28.091	11.097
	SD	1.028	1.504	2.567	0.453	1.222	0.97	1.512	13.966	2.258
	SEM	0.594	0.868	1.482	0.261	0.706	0.56	0.873	8.063	1.304
	Adjusted P Value	0.0155	0.8813	0.3966	0.1147	0.2675	> 0.99	0.1538	0.0317	0.035
	N	3	3	3	3	3	3	3	3	3
Creatinine [10µM] + ritonavir [15µM] compared to untreated condition	Mean	2.813	13.159	6.281	0.839	5.05	1.762	2.413	38.966	25.977
	SD	2.637	7.117	0.691	0.094	5.597	0.789	0.729	53.055	33.358
	SEM	1.522	4.109	0.489	0.054	3.23	0.455	0.421	30.631	23.588
	Adjusted P Value	0.1147	0.0223	0.3521	0.7094	0.8813	0.347	0.1538	0.2039	0.235
	N	3	3	2	3	3	3	3	3	2
Creatinine [10µM] + Cimetidine [100µM]compared to untreated condition	Mean	1.471	5.994	10.546	0.984	3.206	2.077	2.718	16.401	5.061
	SD	0.393	0.485	1.469	0.206	1.482	0.55	0.999	6.108	3.012
	SEM	0.227	0.28	1.039	0.119	0.855	0.318	0.577	3.526	2.13
	Adjusted P Value	0.565	0.0444	0.0209	> 0.99	0.2675	0.2675	0.0616	0.2039	0.7315
	N	3	3	2	3	3	3	3	3	2

Table S4. List of the targeted 103 metabolites.

Metabolites		
2-Ketoisovaleric acid	2-aminobutyric acid	Cytidine monophosphate
Tyrosine	Serine	Cytosine
Histidine	4-Aminobutyric acid	Deoxyadenosine
Thymidine	Aspartic acid	Deoxyadenosine monophosphate
5-Glutamylcysteine	Biotin	Deoxycytidine
Glycyl-glutamine	Arginine	Deoxyguanosine
Threonine	5-Oxoproline	D-Mannitol
Tryptophan	Pyruvic acid	D-Ribose
Folic acid	Aconitic acid	Formylkynurenine
Asparagine	Phenylalanine	Fumaric acid
Adenosine	Isoleucine	Glutathione
Proline	Guanosine	Glycine
Threonic acid	Urocanic acid	Guanine
Gluconic acid	Kynurenic acid	Guanosine monophosphate
1-Methylhistidine	Adenine	Indole-3-acetic acid
Succinic acid	Leucine	Inosine monophosphate
Citric acid	3-Methyl-2-oxovaleric acid	Lysine
Malic acid	Cystine	Methionine
Ornithine	Methionine sulfoxide	N-Acetylaspartic acid
Uric acid	Riboflavin	NAD
Hypoxanthine	Xanthine	Niacinamide
4-Pyridoxic acid	Inosine	Nicotinic acid
2-Aminoadipic acid	Pyridoxal	O-Phosphoethanolamine
Xanthosine	3-aminopropanoic acid	Oxidized glutathione
Alanine	3-hydroxyanthranilic acid	Pipecolic acid
Hexose (Glucose)	4-Hydroxyproline	Putrescine
Citrulline	5'-Methylthioadenosine	Pyridoxal phosphate
Lactic acid	Adenosine monophosphate	Pyridoxine
Uridine monophosphate	Argininosuccinic acid	S-adenosylhomocysteine
Pantothenic acid	Ascorbic acid 2-phosphate	Taurine
Kynurenine	Creatinine	Thymine
Choline	Cystathionine	Uridine
Glutamine	Cysteine	Valine
alpha-keto-glutarate	Cytidine	Xanthosine monophosphate
Glutamic acid		

Table S5. Metabolite expression measured by mass spectrometry under different flow rates.

Mean	Rocker-based perfusion	5 µL/min	10 µL/min	20µL /min
2-Aminoadipic acid	0.07	0.00	0.00	0.00
2-aminobutyric acid	0.83	0.93	0.94	0.95
4-Aminobutyric acid	5.37	6.55	6.43	6.39
4-Pyridoxic acid	0.05	0.05	0.04	0.05
5-Oxoproline	0.08	0.07	0.07	0.06
Adenine	0.21	0.20	0.19	0.19
Alanine	1.16	0.17	0.18	0.18
alpha-keto-glutarate	0.09	0.14	0.14	0.14
Aspartic acid	0.00	0.03	0.03	0.03
Choline	10.54	13.30	13.03	13.13
Citrulline	0.86	1.00	1.00	1.00
Folic acid	0.87	0.68	0.68	0.67
Glutamic acid	1.64	1.24	1.21	1.27
Glutamine	0.23	0.38	0.37	0.36
Guanosine	0.03	0.03	0.03	0.04
Hexose (Glucose)	0.11	0.15	0.15	0.16
Histidine	1.56	1.54	1.53	1.52
Hypoxanthine	0.00	0.23	0.23	0.24
Isoleucine	19.39	19.50	19.03	19.06
Kynurenic acid	0.30	0.31	0.30	0.30
Lactic acid	22.73	1.06	1.07	1.00
Leucine	3.69	4.03	3.97	3.97
Malic acid	0.06	0.00	0.00	0.00
Methionine sulfoxide	0.10	0.11	0.11	0.11
Phenylalanine	40.64	40.27	40.19	40.30
Proline	9.39	8.48	8.37	8.43
Pyruvic acid	0.52	0.49	0.50	0.49
Riboflavin	0.49	0.55	0.55	0.52
Thymidine	0.04	0.03	0.03	0.03
Tyrosine	11.90	11.07	11.02	10.99
Uridine monophosphate	0.01	0.04	0.04	0.04
Xanthine	0.03	0.00	0.00	0.00
Xanthosine	0.02	0.00	0.00	0.00

Table S6. Metabolite dosage on RPTEC/TERT1 under metformin or creatinine treatment compared to untreated condition. The cells are cultured in mono-channel device under 20 µL/min flow.

Metabolite	Metformin	Creatinine
2-aminobutyric acid	0.80	0.97
5-Oxoproline	0.43	1.61
Adenine	0.98	1.06
Adenosine	0.89	0.60
Alanine	0.84	0.95
alpha-keto-glutarate	0.53	0.75
Arginine	1.06	0.79
Asparagine	0.78	0.65
Aspartic acid	0.65	0.65
Choline	0.81	0.96
Citrulline	0.97	0.79
Creatinine	0.31	na
Cystine	0.95	0.52
Folic acid	1.09	1.18
Glutamic acid	0.88	0.96
Glutamine	0.50	0.76
Guanosine	0.63	0.70
Hexose (Glucose)	1.03	0.79
Histidine	0.90	0.68
Hypoxanthine	0.96	1.08
Isoleucine	0.77	1.04
Lactic acid	0.83	1.95
L-Carnitine	0.65	0.68
Leucine	0.67	1.04
Methionine sulfoxide	1.14	1.27
Niacinamide	0.79	0.95
Ornithine	0.71	0.38
Pantothenic acid	0.45	1.10
Phenylalanine	0.93	1.08
Pipecolic acid	0.34	1.32
Proline	0.82	0.83
Pyruvic acid	0.94	1.11
Riboflavin	0.97	1.03
Serine	0.85	0.64
Threonine	1.03	0.83
Thymine	1.15	0.88
Tryptophan	1.04	1.11
Tyrosine	1.05	1.09

Supplementary Figures

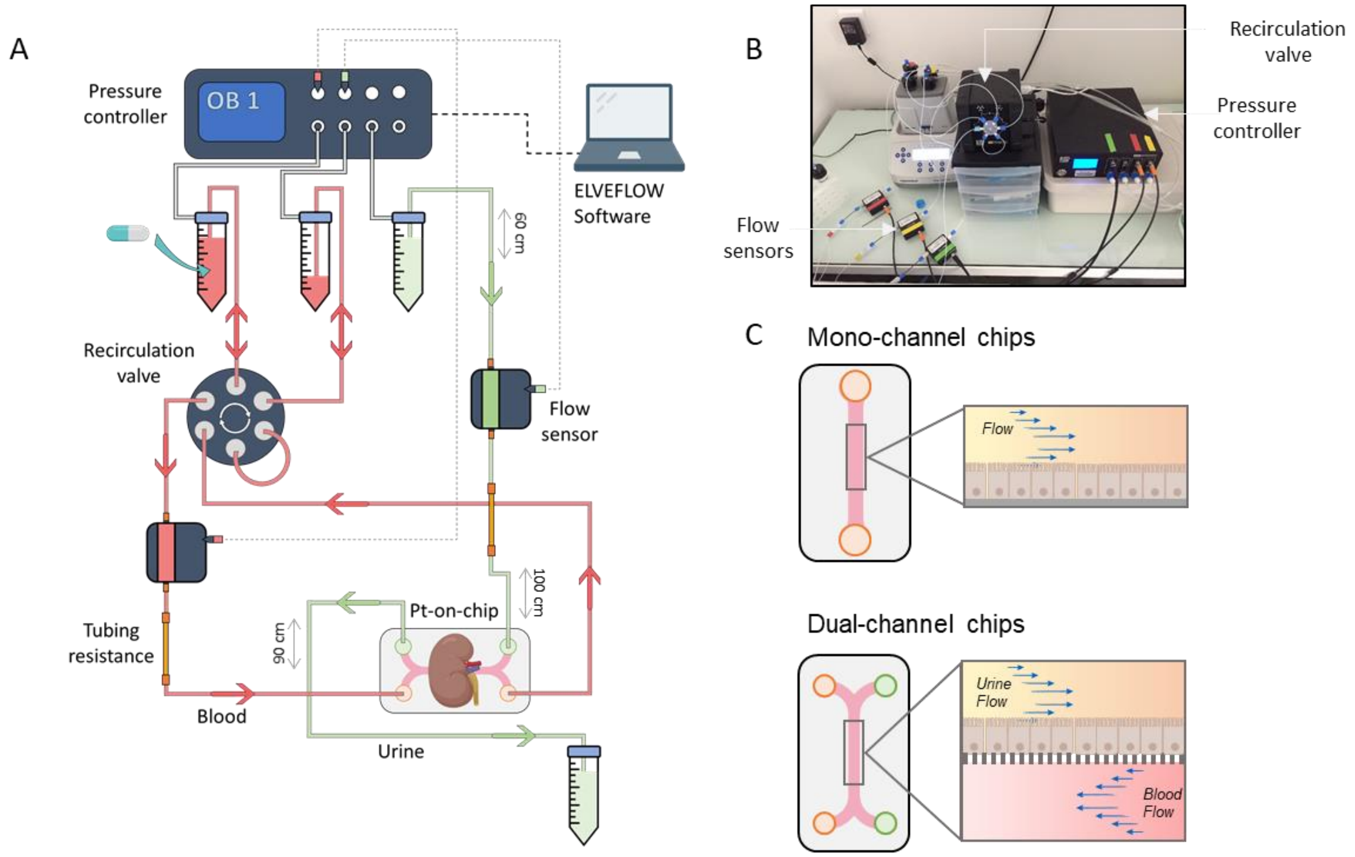

Figure S1. Overview of commercial microfluidics setup and devise. (A) Our microfluidic system consists of a pressure controller who will administered pressure to the system which will ensure the flow. The flow rate it's measure by flow sensors and controlled by the elveflow ™ software. The tubbing resistance permit much more precise control of the flow rates.(B) lab view of the microfluidic system. (C) Schematic view of commercial mono (ibidi ™) and dual (beonchip ™) channel device use for RPTEC/TERT1 culture.

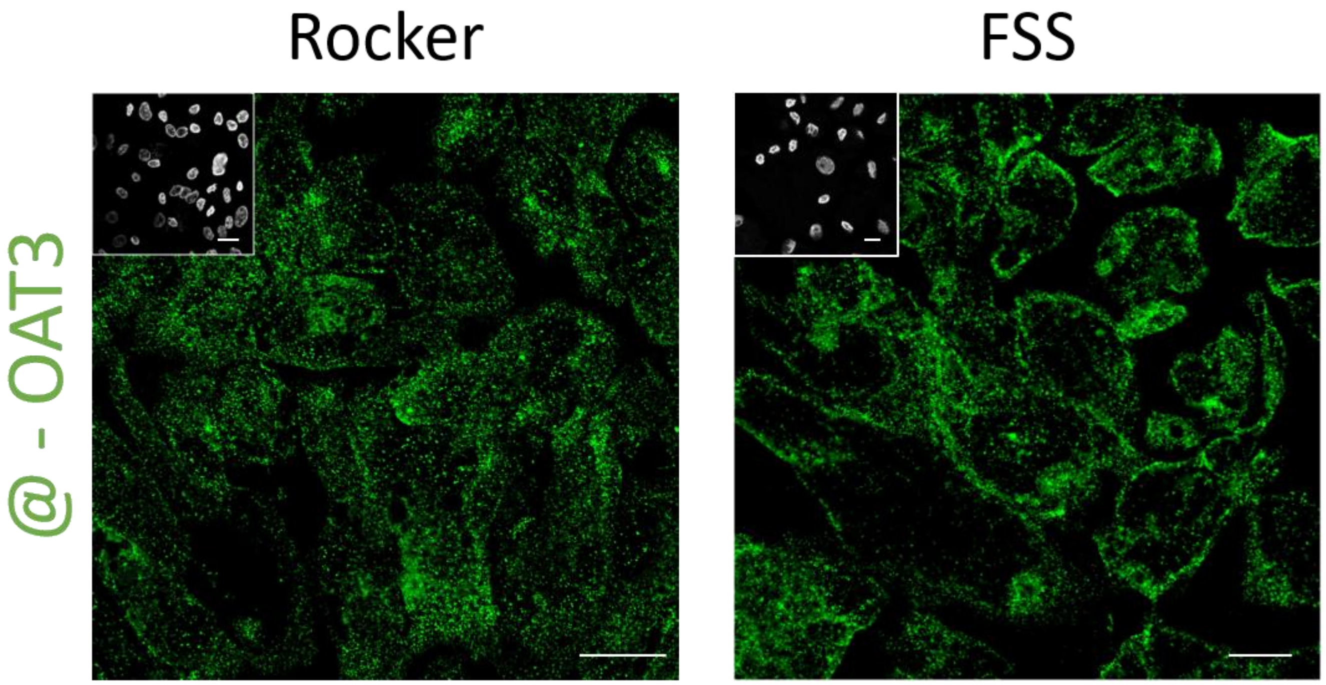

Figure S2. Immunolabeling of OAT3 on RPTEC/TERT1. Cells are cultured under rocker-based perfusion (Rocker) or FSS (20 µL/min flow; 0.02 dyn/cm²) condition. Insert represent DAPI staining, scale bar represents 20µM.

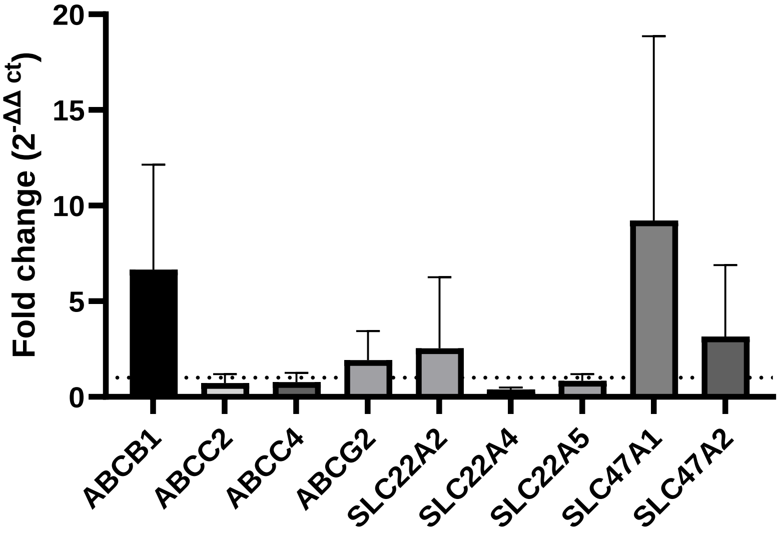

Figure S3. Fold changes regarding the relative expression levels of mRNA transporter expressions of RPTEC/TERT1 cell line in dual-channel device under 10 µL/min (0.03 dyn/cm²) in apical compartment and 20 µL/min (0.07 dyn/cm²) in basal compartment (N=4). Error bars represent standard deviations. Statistical significance was determined using Kruskal Wallis test, asterisks (*) indicate statistically significant differences with respect to mono-channel device under 20 µL/min (0.02 dyn/cm², N=4), *p-value < 0.05, **p-value < 0.01, ***p-value < 0.001).

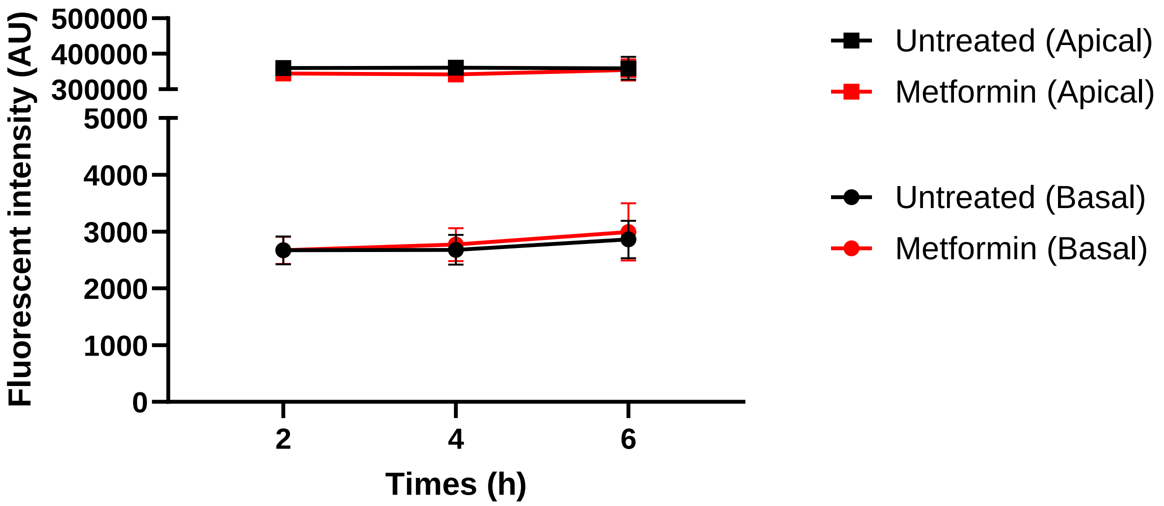

Figure S4. FITC-dextran permeability assay. The permeability of the cell layer was assessed by measuring the rate of fluorescein isothiocyanate (FITC)-dextran passage in presence or not of metformin 100µM, from the apical to the basal compartment of a transwell system. 20µM of FITC-conjugated 10kDA dextran (FITC-dex, Sigma-Aldrich) was introduced into the apical compartment and the FITC-dextran transport across the barrier was determined by serially sampling fluid from the apical and basal compartment and measuring its fluorescence intensity, which was used as an index of barrier permeability.

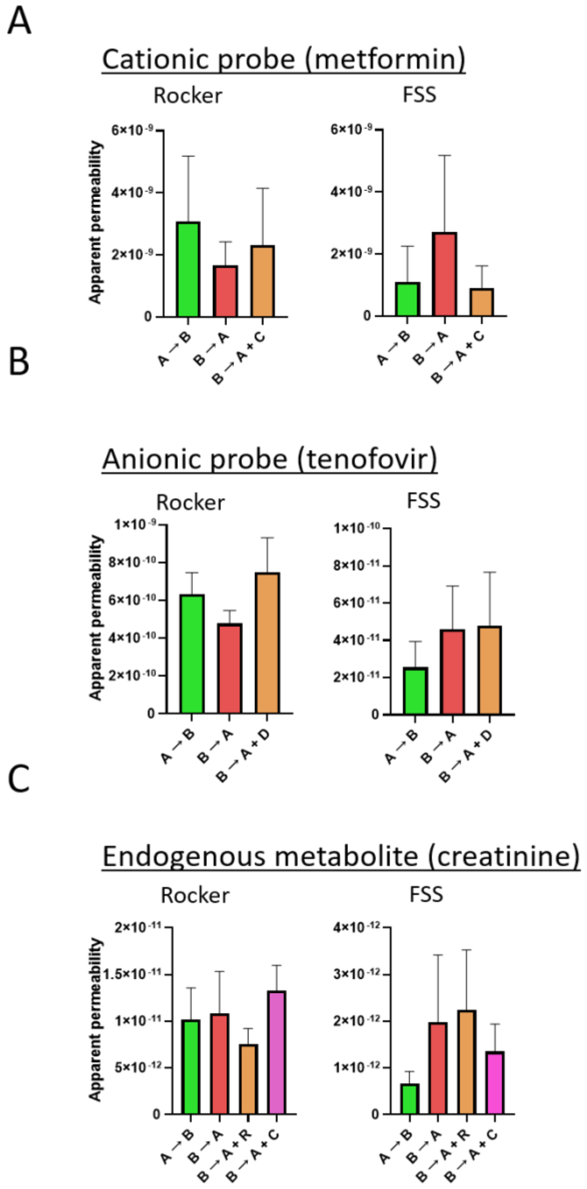

Figure S5. Calculated apparent permeabilities for transcellular transport. Apparent permeabilities were calculated for (A) metformin (100 μM, N=4), (B) tenofovir (30 μM, N=4) and (C) creatinine (10 μM, N=8) as cationic, anionic and endogenous probes, respectively. P_{app} were calculated considering transcellular transport from apical to basal compartment (A→B) and basal to apical compartment (B→A) for rocker-based perfusion (Rocker) and FSS conditions in presence or not of an inhibitor, namely cimetidine (C, 100 μM, N=3) for metformin; diclofenac (D, 30 μM, N=4) for tenofovir and ritonavir (R, 15 μM, N=3) or cimetidine (C, 100 μM, N=3) for creatinine.
